# Incident Crohn’s Disease as a Risk Factor for Colorectal Cancer in the First 10 Years after Diagnosis: A Nationwide Population-Based Study

**DOI:** 10.3390/jcm10204663

**Published:** 2021-10-12

**Authors:** Hyunil Kim, Ji Hoon Kim, Jung Kuk Lee, Dae Ryong Kang, Su Young Kim, Hyun-Soo Kim, Hee Man Kim

**Affiliations:** 1 Department of Internal Medicine, College of Medicine, Yonsei University Wonju, Wonju 26426, Korea; kimhyunil@empal.com (H.K.); breeze1212@yonsei.ac.kr (S.Y.K.); 2 Department of Medicine, College of Medicine, Yonsei University Wonju, Wonju 26426, Korea; jh12111000@hanmail.net; 3 Center of Biomedical Data Science, College of Medicine, Yonsei University Wonju, Wonju 26426, Korea; dlwjdrnr23@naver.com (J.K.L.); dr.kang@yonsei.ac.kr (D.R.K.)

**Keywords:** colorectal cancer, Crohn’s disease, risk factors, late-onset

## Abstract

We investigated the risk of colorectal cancer (CRC) in patients with Crohn’s disease (CD) using the claims data of the Korean National Health Insurance during 2006–2015. The data of 13,739 and 40,495 individuals with and without CD, respectively, were analyzed. Hazard ratios (HRs) were calculated using multivariate Cox proportional hazard regression tests. CRC developed in 25 patients (0.18%) and 42 patients (0.1%) of the CD and non-CD groups, respectively. The HR of CRC in the CD group was 2.07 (95% confidence interval (CI), 1.25–3.41). The HRs of CRC among men and women were 2.02 (95% CI 1.06–3.87) and 2.10 (95% CI, 0.96–4.62), respectively. The HRs of CRC in the age groups 0–19, 20–39, 40–59, and ≥60 years were 0.07, 4.86, 2.32, and 0.66, respectively. The HR of patients with late-onset CD (≥40 years) was significantly higher than that of those with early-onset CD (<40 years). CD patients were highly likely to develop CRC. Early-onset CD patients were significantly associated with an increased risk of CRC than matched individuals without CD. However, among CD patients, late-onset CD was significantly associated with an increased risk of CRC.

## 1. Introduction

Inflammatory bowel disease (IBD) is a group of disorders that cause chronic inflammation in the gastrointestinal tract, including Crohn’s disease (CD) and ulcerative colitis (UC). CD is associated with an increased risk of colorectal cancer (CRC). Although a previous meta-analysis showed more reduced incidence rates of CRC among IBD patients in the 2000s than for those in the 1990s, the risk of CRC in patients with CD remains higher than that of the background population [1].

The incidence rate of IBD in Asian countries, including South Korea, has progressively increased, with the rate of CD exhibiting a steeper increment than that of UC [2]. A cohort study from South Korea showed that the mean annual incidence rate of CD increased significantly from 0.06 per 100,000 inhabitants in 1986–1990 to 2.44 per 100,000 inhabitants in 2011–2015 [3].

Considering that the course of CD is greatly affected by the development of new treatments and drugs, the risk of CD-associated CRC is also expected to change. Therefore, it is important to understand the risk of CRC in patients who have newly developed CD in relatively recent periods, especially in South Korea where the incidence of CD has been increasing.

Therefore, we conducted a population-based study to assess the updated risk of CRC in incident CD by comparing the information of age- and sex-matched individuals available from the claims data of the Korean National Health Insurance (NHI) between 2006 and 2015.

## 2. Materials and Methods

### 2.1. Study Population

We designed a cohort study to estimate the risk of development of CRC in CD patients. The data for our study were obtained from the NHI database comprising the claims data of medical aid and NHI beneficiaries [4]. The NHI database contains comprehensive information on beneficiaries, including demographic characteristics, treatment claims for hospitalization, and ambulatory care diagnoses based on disease classification according to the International Classification of Diseases, 10th revision (ICD-10), pharmaceutical prescriptions, and procedures [5].

We have done research on UC and CRC, and we have already published the results [4]; the present study is similar in terms of the research method. The details on the methodology of the present study are described below.

For this retrospective study, the cohort comprised patients with CD and matched reference individuals without CD selected from the Korean nationwide population. CD was defined using the ICD-10 diagnostic code (K50) and prescription for IBD. Patients who received a diagnosis of CD between January 2004 and December 2015 were selected from the medical aid and NHI beneficiaries. A washout period of 2 years (January 2004–December 2005) was then considered to exclude prevalent cases because we hypothesized that patients with CD would have visited the hospital at least once within two years. Therefore, patients who had not visited the hospital during the set washout period and subsequently visited the hospital with a diagnosis of CD, as defined in this study, were considered to have newly diagnosed incident CD. Eventually, patients with incident CD that was diagnosed between January 2006 and December 2015 were enrolled from among 957,056,482 beneficiaries of Medical Aid and NHI. For the non-IBD reference group, age- and sex-matched individuals without the diagnostic codes of IBD (K50 for CD and K51 for UC) were selected randomly at a 3:1 ratio from the NHI database between January 2006 and December 2015. The primary endpoint was an incidence of CRC. A stratified random sampling was used when selecting the matched individuals. In other words, stratification according to age, gender, region, and insurance quantile was divided and random sampling was performed for each stratification.

### 2.2. Definition

The operational definitions were retrieved from the NHI data, mainly based on the insurance claims data, to identify newly diagnosed CD and CRC cases for data analysis. Patients with CD were defined as those with the ICD-10 diagnosis code K50 and records of prescription for CD. We defined the prescriptions for CD as use of (1) steroids for 3 months; (2) 5-aminosalicylic acid; (3) immunomodulators, such as azathioprine, 6-mercaptopurine, and/or methotrexate at least once; and/or (4) biologic agents, such as tumor necrosis factor-alpha antagonist, at least once. Our operational definitions revealed that sensitivity was 93.1% (91–94.7) and specificity was 98.1% (96.9–98.8), according to a study for validation of diagnosis of IBD in South Korea [6].

Patients with CRC were defined as those with ICD-10 diagnosis codes C18, C19, and C20. We additionally used the V code system as part of the effort to exclude false-positive CRC. The V code is specific to South Korea, which has been developed for registering patients with “rare and intractable disorders/diseases (RID)” to the Korean NHI [7]. This system is intended to provide economic compensation to RID-registered patients, while a significant portion of medical expenses is reduced. The V code system tries to exclude clinically suspicious diagnosis of diseases, and it accepts pathological and radiological confirmative diagnosis. The V code is much more accurate than the ICD code for cancer classification. Consequently, it is used in many big data studies in Korea. In our study, we tried to exclude false-positive CRC using the V code. The V codes for colorectal cancer (C18-20) are V193–194. Patients with CD and reference individuals who did not fulfill the operational definition of CRC during the study periods were censored on the date of dropout (due to death or emigration) or at the end date of follow-up (December 2015).

### 2.3. Statistical Analysis

The standardized incidence ratio (SIR) is the ratio of the observed number of cancer cases to the expected number of cases. It was calculated to demonstrate whether patients with CD had a higher risk of CRC compared to the general population. The number of incident cancer cases was considered the number of observed cases. We calculated the expected number of cases for different age groups (5-year intervals) using information on the incidence rate of cancer among the general population obtained from the National Cancer Registry. The expected number of cancer cases was computed by multiplying the age-specific incidence rate of the general population from 2011 (as reported by the National Cancer Registry) by the person-years of patients with CD. We calculated the 95% confidence intervals (CIs) of the SIR using Byar’s approximation when the number of observed cases was ≥10 and chi-squared distribution when the number of observed cases was 1–9 [8]. Microsoft Office Excel 2007 (Microsoft Corporation, Redmond, Washington, DC, USA) and SAS program, version 9.4 (SAS Institute Inc., Cary, NC, USA), were used to perform the statistical analyses.

Comorbidities, socioeconomic status, residence, and modified Charlson Comorbidity Index (CCI) score were included as covariate variables to evaluate the risk of CRC. The following comorbidities (ICD-10 codes) were considered: hypertension (I10-13, I15), diabetes mellitus (E78), cholangitis (K83), anal fistula (K60.3), cerebral vascular disease (I60-69), and cardiovascular disease (I20-25, I34-37). The CCI score was calculated to predict 1-year mortality by categorizing comorbidities based on ICD codes [9,10]. We excluded tumor factors when calculating the CCI score because CRC was the primary endpoint of our study. The socioeconomic status was defined as the health insurance quantile according to the health insurance cost. The quantile is the 20th quartile, and the quantile is divided into thirds (low, mid, high).

Continuous variables were presented as means ± standard deviations and categorical variables as numbers and percentages. To compare characteristics between the groups, Student’s *t*-test for continuous variables and the chi-squared test for binary and categorical variables were used. Multivariate Cox regression models with Firth correction were used to assess the risk of CRC in CD patients using age- and sex-matched individuals as references. The hazard ratios (HRs) of CRC were adjusted for age, sex, socioeconomic status, residence, and comorbidities, including hypertension, diabetes mellitus, cholangitis, anal fistula, cerebrovascular disease, and cardiovascular disease. All statistical tests were two-tailed, and a *p*-value < 0.05 was considered statistically significant. All analyses were performed using SAS software, version 9.4 (SAS Institute Inc., Cary, NC, USA).

## 3. Results

### 3.1. Study Population

**Figure 1:**
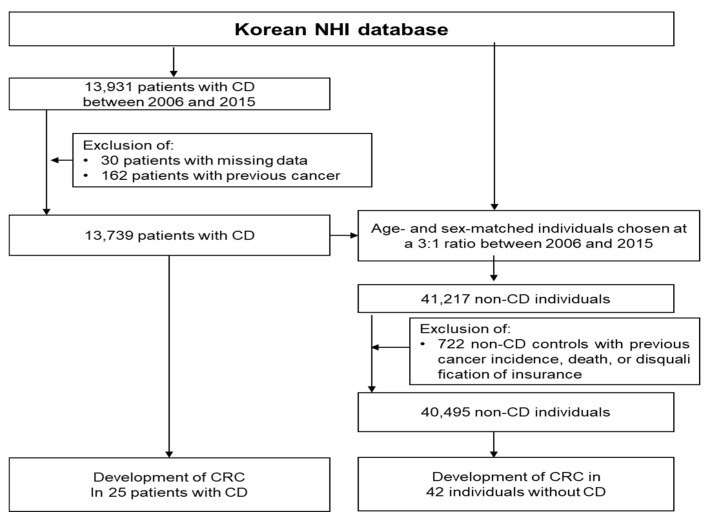
Flow chart of the study population. NHI, National Health Insurance; CD, Crohn’s disease; CRC, colorectal cancer.

Between 2006 and 2015, 13,931 patients were identified as having newly diagnosed CD, of which 192 were excluded (30 patients with missing data and 162 who previously had cancer). In total, 13,739 patients were enrolled in the CD group. A total of 41,217 age- and sex-matched individuals were selected as controls from the general population, among whom 722 were excluded because of previous cancer incidence, death, or disqualification of insurance. Thus, 40,495 matched individuals were enrolled in the non-CD group (Figure 1).

The CD and non-CD groups were analyzed for mean follow-up periods of 4.77 ± 2.88 years and 4.84 ± 2.88 years, respectively. The proportion of men in the CD group was 71.01% (9756 men), which was similar to that in the non-CD group (71.12%). The mean age of the CD group was 29.6 ± 14.9 years, which was slightly higher than that of the non-CD group (29.3 ± 14.7 years). Higher proportions of individuals in the CD group exhibited a high socioeconomic status and resided in urban areas, as opposed to those in the non-CD group (*p* < 0.001). The CD group had a higher proportion of diabetes mellitus, cerebrovascular disease, cardiovascular disease, cholangitis, and anal fistula than the non-CD group (Table 1).

### 3.2. Incidence of CRC among Patients with CD

In the CD group, 25 patients (15 men, 10 women) developed CRC while 42 patients from the non-CD group developed CRC (Table 2). The crude incidence rate of CRC was 38.2 per 100,000 persons in the CD group, and it was 21.4 per 100,000 persons in the non-CD group (Table 1). The SIRs of CRC in the CD and non-CD groups were 1.49 (95% CI, 0.83–2.46) and 0.84 (95% CI, 0.54–1.24) for men, respectively. Among women, the SIRs of CRC in the CD and non-CD groups were 1.93 (95% CI, 0.92–3.55) and 1.08 (95% CI, 0.63–1.73), respectively (Table 2).

### 3.3. Risk of CRC in CD Patients Stratified by Sex and Age

**Figure 2:**
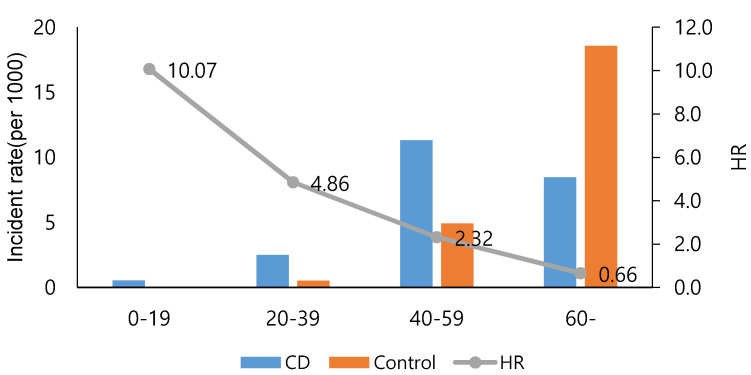
Comparison of incidence rate and HR of CRC between CD and non-CD groups based on age. HR, hazard ratio; CD, Crohn’s disease; CRC, colorectal cancer.

The CD group exhibited significantly higher risk of CRC than the non-CD group (HR, 2.07; 95% CI, 1.25–3.41). The HRs of men and women in the CD group were 2.02 (95% CI, 1.06–3.87) and 2.10 (95% CI, 0.96–4.62), respectively (Table 3). Figure 2 shows the stratification of HR by age when CD was diagnosed. Patients ≥60 years in the CD group had a lower incidence of CRC than those in the non-CD group, whereas the patients belonging to other age categories (0–19, 20–39, and 40–59 years) of the CD group showed a higher incidence of CRC than those in the non-CD group. In the CD group, the HRs of children and adolescents (0–19 years), young adults (20–39 years), and middle-aged individuals (40–59 years) were 10.07, 4.86, and 2.32 respectively, which were higher than those of the non-CD group. However, the HR was 0.66 in the oldest group (≥60 years), which was lower than that of this age group in the non-CD group.

Among the men in the CD group, the HR of the 0–39 age group was 4.02 (95% CI, 1.05–15.36) and that of the ≥40 age group was 1.60 (95% CI, 0.75–3.42). For women in the CD group, the HR of the 0–39 age group was 9.67 (95% CI, 1.20–78.13) and that of the ≥40 age group was 1.34 (95% CI, 0.53–3.40) (Table 4).

### 3.4. Risk Factors for CRC among Patients with CD

We subdivided the CD group into CRC and non-CRC groups to investigate the risk factors for CRC in patients with CD (Table 5). The patients in the CRC group received a CD diagnosis at an older age than those in the non-CRC group. Multivariable analyses after adjusting for covariates showed that persons with an older age of diagnosis (≥40 years) had significantly higher HR for CRC than those in whom CD was diagnosed at a younger age (0–19 years). Other variables, including sex, were not significant (Table 6).

## 4. Discussion

For nearly a century, it has been known that there is an association between IBD and CRC, which is assumed to be promoted by a chronic inflammation-driven carcinogenic process in the intestine [11]. Although the entire underlying mechanisms remains unclear, inflammatory mediators produced in the chronic inflammatory process may contribute to the development of CRC [11]. CD is a known risk factor for CRC [12]. Previous meta-analyses of population-based cohort studies proved that patients with CD are at an increased risk of intestinal cancer [13,14].

Our nationwide Korean cohort study showed that the crude incidence rate of CRC in the CD group was higher than that of CRC in the non-CD group (0.38/1000 person-years vs. 0.21/1000 person-years). Furthermore, the CD group had a higher relative risk of CRC than the non-CD group (adjusted HR, 2.07; 95% CI, 1.25–3.41); these findings are consistent with a previous Korean study that showed CD increases the risk of CRC [5]. According to the Korea Central Cancer Registry database, the baseline incident cases of CRC in the Korean population during 2006–2015 were 258,051, and the crude incidence rate of CRC in Korean population during 2006–2015 was 51.7 per 100,000 persons.

Additionally, in our study, stratification by age at which CD was diagnosed showed an increased risk of CRC in younger CD patients. This finding is similar to that of a previous cohort study in which 47,374 Danish IBD patients were followed for 30 years [15]. Although the reason for the difference in CRC risk between different age groups was not clear, it could be attributed to the relatively sharp rise in the incidence of CRC among older persons in the non-CD group (Figure 2).

Further analysis of the characteristics of CD patients categorized based on CRC occurrence showed the HR of late-onset CD (≥40 years old) was higher than that of early-onset CD (<40 years old) (Table 6). Thus, late-onset CD (≥60 years) may be an independent risk factor for CRC (Figure 2). In South Korea, the age-standardized incidence rate of colon cancer is higher than that in the US [16], which could be because of increased detection through the national CRC screening program [17].

The research period for our study is 10 years (2006–2015), which is relatively short. Recently, with the advent of biologics, the disease course has changed dramatically and the diagnostic equipment has been developed more precisely, so we wanted to know how this affects the occurrence of colorectal cancer. Since we wanted to reflect the current situation, we used a relatively recent period. Usually, duration and severity of inflammation in CD are important risk factors for colorectal cancer. However, even during our short study period, CRC was more common in CD patients than in the general population. We do not know why CRC occurs more frequently in CD patients than in normal people in a short period of time within 10 years of diagnosis. However, such a phenomenon has also been observed in another study [15].

There are several specificities in CD and CRC in Korea [18]. The location of CRC in CD patients is different between Korea and Western countries: 92% of CRC occurs in the rectum of CD patients in Korea [19], but 40% of CRC occurs in the rectum in the West [1]. In Korea, this may be due to a high prevalence of perianal fistula, which is associated with a cumulative probability of rectal cancer in CD [19]. Incidence of perianal fistula is very high in Korean patients with CD. One hospital-based study in Korea showed that about 50% of Korean patients experienced perianal fistula during the median follow-up period of 4 years [20], as compared to 13–38% in Western countries [21]. The SIR of CRC in CD is higher than in UC (6.0 vs. 1.7), inconsistent with Western countries [19]. This difference may be partly due to the age discrepancy at diagnosis in Korea [18].

Additionally, in Korea, 5-ASA prescription rates are very high. The Korean Association for the Study of Intestinal Disease (KASID) multicenter study of 728 CD patients reported that 98.1% of patients were prescribed 5-aminosalicylate (5-ASA), and 59.5% and 18.0% were prescribed oral or intravenous corticosteroids, respectively. Thiopurine drugs were used in 65.0% and infliximab was used in 26.9% of patients [22]. In Korea, only step-up treatment is covered by health insurance. Top-down is not covered by insurance. Therefore, doctors may prescribe 5-ASA as an initial therapy.

Our study included children who have low incidence of CRC, particularly within 10 years after diagnosis of CD. However, CRC can occur in children with CD. One Danish nationwide cohort study showed that the relative risk of pediatric CD patients with 0–19 years was 43.8 (95% CI: 27.2–70.7) [15]. Additionally, CRC can develop in relatively short periods after diagnosis of CD. The other population-based study in Denmark and Finland showed that, among IBD patients (<18 years old), the median time from IBD diagnosis to cancer was 11.2 years (range: 0.04–19.3) [23].

The strength of our study lies in the fact that in this large-scale, nationwide study, the cohort that was stratified by age and sex may be representative of the general IBD population of South Korea. However, our study has some limitations, mainly related to the NHI data. Firstly, the diagnosis of CD was based on an operational definition with ICD codes and prescription. However, since IBD is mostly diagnosed and treated at tertiary hospitals by IBD specialists in South Korea, the ICD codes of IBD are considered correct [24]. Secondly, because NHI does not have detailed medical records, we did not consider other factors of CD, such as disease severity or the site involved (ileal, ileocolic, anorectal), which are associated with CRC risk. Thirdly, this study is retrospective with no data from the prescription period; therefore, we were unable to explain the relevance between IBD medications and CRC development. Lastly, the location of CRC in CD is a very important confounding factor, but we did describe CRC location. We did not discriminate the location of CD in the disease code when extracting CD patients from the NHI database because the number of colorectal cancer patients was so small in CD. In the next study, if the number of colorectal cancer patients is further increased by extending the study period to 30 years, it will be possible to analyze the location of the colon as well.

In conclusion, the risk of CRC is higher in incident CD patients than in the general population. Early-onset CD patients exhibit higher risk for CRC than matched individuals without CD, while among the CD patients, late-onset CD is significantly associated with an increased risk of CRC than early-onset CD.

## Figures and Tables

**Table 1:** Baseline characteristics of the study population.

Characteristic	CD Patients	Matched Non-CD Individuals	*p*-Value
Total number	13,739	40,495	
Person-years	65,505	196,132	
Colorectal cancer	25 (0.18)	42 (0.10)	
Incidence rate (/1000 person-years)	0.38	0.21	
Sex (male)	9756 (71.01)	28,802 (71.12)	0.7967
Age at index date			
Mean ± SD (years)	29.6 ± 14.9	29.3 ± 14.7	0.0281
0–19 years	4229 (30.78)	12,665 (31.28)	0.2072
20–39 years	6497 (47.29)	19,220 (47.46)	
40–59 years	2247 (16.35)	6518 (16.10)	
≥60 years	766 (5.58)	2092 (5.17)	
Social economic status			<0.0001
Low	3291 (23.95)	10,579 (26.12)	
Mid	4127 (30.04)	14,893 (36.78)	
High	6321 (46.01)	15,023 (37.10)	
Residence			<0.0001
Urban	6757 (49.18)	17,461 (43.12)	
Suburban	6098 (44.38)	17,958 (44.35)	
Rural	874 (6.36)	4028 (9.95)	
Comorbidities			
Hypertension	1797 (13.08)	4993 (12.33)	0.0218
Diabetes Mellitus	2344 (17.06)	4319 (10.67)	<0.0001
Cerebrovascular disease	98 (0.71)	171 (0.42)	<0.0001
Cardiovascular disease	107 (0.78)	174 (0.43)	<0.0001
Cholangitis	329 (2.39)	243 (0.60)	<0.0001
Anal fistula	3856 (28.07)	365 (0.90)	<0.0001
Modified CCI score *			<0.0001
0	5156 (37.53)	19,338 (47.75)	
1	4979 (36.24)	13,728 (33.90)	
2	2140 (15.58)	4611 (11.39)	
≥3	1464 (10.66)	2818 (6.96)	
IBD medication			
Steroid	7240 (52.70)	1602 (3.96)	<0.0001
5-ASA	13,310 (96.88)	6 (0.01)	<0.0001
Thiopurines	9063 (65.97)	26 (0.06)	<0.0001
Biologics	3437 (25.02)	3 (0.01)	<0.0001
Follow-up period (years)	4.77 ± 2.88	4.84 ± 2.88	0.0080

Data are presented as a number (%). CD, Crohn’s disease; CCI, Charson Comorbidity Index; 5-ASA, 5-aminosalicylic acid. * Modified CCI score is defined as CCI score without tumor factor.

**Table 2:** Standardized incidence ratio of colorectal cancer in CD patients.

	CD				Non-CD Controls			
	Observed, N	Expected, N	SIR	95% CI	Observed, N	Expected, N	SIR	95% CI
Male	15	10.07	1.49	0.83–2.46	25	29.74	0.84	0.54–1.24
Female	10	5.19	1.93	0.92–3.55	17	15.73	1.08	0.63–1.73

CRC, colorectal cancer; SIR, standardized incidence ratio; CI, confidence interval.

**Table 3:** Hazard ratio of CD for CRC by sex.

	CD			
	CRC Cases	Adjusted HR	95% CI	*p* Value
Total	25	2.07	1.25–3.41	0.0045
Male	15	2.02	1.06–3.87	0.0329
Female	10	2.10	0.96–4.62	0.0646

**Table 4:** Hazard ratio of CD for CRC by sex and age.

Sex	Age at Diagnosis (Year)	CD			
		CRC Cases	Adjusted HR	95% CI	*p* Value
Male	0–39	5	4.02	1.05–15.36	0.0423
	≥40	15	1.60	0.75–3.42	0.2227
Female	0–39	4	9.67	1.20–78.13	0.0334
	≥40	6	1.34	0.53–3.40	0.5422

HR, hazard ratio; CRC, colorectal cancer. Multiple analysis including age, sex, social economic status, residence, hypertension, diabetes mellitus, cerebral vascular disease, and cardiovascular disease.

**Table 5:** Characteristics of CD patients by CRC.

Characteristic	CD Patients		
	CRC	Non-CRC	*p*-Value
Total number	25	13,714	
Person-years	81	65,424	
Sex (male)	15 (60)	9741 (71.03)	0.2246
Age at diagnosis of CD			
Mean ± SD	45.2 ± 15.8	29.6 ± 14.9	<0.0001
<40	9 (36)	10,717 (78.15)	<0.0001
≥40	16 (64)	2997 (21.85)	
Social economic status			0.1713
Low	8 (32)	3283 (23.94)	
Mid	9 (36)	4118 (30.03)	
High	8 (32)	6313 (46.03)	
Residence			0.2232
Urban	10 (40)	6747 (49.20)	
Suburban	12 (48)	6086 (44.38)	
Rural	3 (12)	871 (6.35)	
Comorbidities			
Hypertension	6 (24)	1791 (13.06)	0.1285
Diabetes Mellitus	6 (24)	2338 (17.05)	0.4201
Cerebral vascular disease	0 (0)	98 (0.71)	1.0000
Cardiovascular disease	0 (0)	107 (0.78)	1.0000
Cholangitis	0 (0)	329 (2.40)	1.0000
Anal fistula	5 (20)	3851 (28.08)	0.5047
* Modified CCI score			0.1429
0	10 (40)	5146 (37.52)	
1	3 (12)	4976 (36.28)	
2	7 (28)	2133 (15.55)	
≥3	5 (20)	1459 (10.64)	
IBD medication			
Steroid	11 (44)	7229 (52.71)	0.4266
5-ASA	22 (88)	13,288 (96.89)	0.0418
Thiopurines	9 (36)	9054 (66.02)	0.0025
Biologics	5 (20)	3432 (25.03)	0.6514
Follow-up period, year	3.24 ± 3.05	4.77 ± 2.88	0.0079

Data are presented as a number (%). CD, Crohn’s disease; IBD, inflammatory bowel disease; CCI, Charson Comorbidity Index; 5-ASA, 5-aminosalicylic acid. * Modified CCI score is defined as CCI score without tumor factor.

**Table 6:** Multi-variate analysis of risk factors of CRC in CD patients.

	CD		
	HR *	95% CI	*p*-Value
Sex			
Male	1		
Female	1.15	0.51–2.61	0.7410
Age at diagnosis			
<40	1		
≥40	8.02	3.06–21.02	<0.0001
Social economic status			
Low	1		
Mid	0.91	0.35–2.37	0.8508
High	0.47	0.18–1.26	0.1342
Residence			
Urban	1		
Suburban	1.32	0.57–3.09	0.5180
Rural	2.20	0.63–7.66	0.2147
Comorbidities			
Hypertension	0.79	0.28–2.20	0.6460
Diabetes Mellitus	0.79	0.29–2.11	0.6349
Cerebral vascular disease	1.03	0.06–17.39	0.9830
Cardiovascular disease	1.18	0.08–17.93	0.9050
Cholangitis	0.61	0.04–9.99	0.7277
Anal fistula	1.42	0.49–4.12	0.5227

* Multiple analysis including age, sex, social economic status, residence, hypertension, diabetes mellitus, cerebral vascular disease, cardiovascular disease, cholangitis, anal fistula.

## Data Availability

Not applicable.
